# Supplementary material for: FGFR1 Inhibition by Pemigatinib Enhances Radiosensitivity in Glioblastoma Stem Cells Through S100A4 Downregulation
Source: Cells. 2025 Sep 11;14(18):1427. doi: 10.3390/cells14181427 (PMC12468751; doi:10.3390/cells14181427)
Supplement: Supplementary file 1 [file cells-14-01427-s001.zip › cells-3666949-supplementary.pdf]

Supplementary Materials

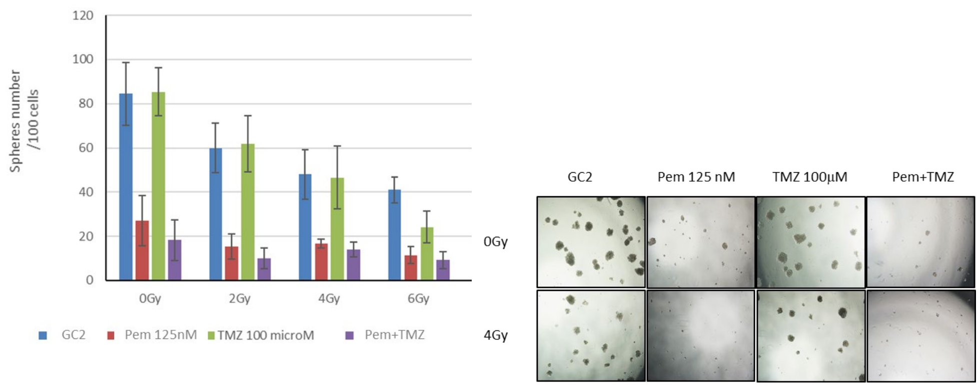

Figure S1. Effect of pemigatinib in association with standard treatments on GC2 glioblastoma stem-like cells. Sphere formation was assessed by clonogenic assay after GC2 cells were treated with pemigatinib (125 nM), temozolomide (100 µM), and irradiation (2–6 Gy). Bar graphs represent the mean ± SD of sphere numbers per 100 cells from descriptive replicate experiments in GC2 cells only; no statistical analysis was performed. Photomicrographs correspond to representative clonogenic assays at 4 Gy, illustrating the observed trends.

Table S1. FGFR1, FGFR2, and FGFR3 mutations identified in GC1 and GC2 cell lines. This table summarizes the mutations detected in the FGFR1, FGFR2, and FGFR3 genes in GC1 and GC2 primary stem cell lines derived from glioblastoma specimens. The listed mutations include gene name, nucleotide change, amino acid substitution, and mutation type, including single nucleotide polymorphisms (SNPs) and variants of uncertain significance (VUS), as identified through sequencing analyses.

	#Uploaded_variation	EXON	CDS_position	Protein_position	Protein	Alamut/COSMIC/gnomAD/OncoKB	Consequence	SYMBOL	Feature_type	MANE_SELECT
GC2_FGFR1	chr8:38 426 236_C/T (G>A)	06/18	631	211	p.Ala211Thr	Variant of unknown significance **(VUS)**	coding_sequence_variant	FGFR1	Transcript	NM_023110.3
GC2_FGFR3	chr4:1 799 513_G/A	03/18	369	123	p.Val123Val	SNP	coding_sequence_variant	FGFR3	Transcript	NM_000142.5
GC2_FGFR3	chr4:1 801 491_G/C	05/18	570	190	p.Lys190Asn	VUS	coding_sequence_variant	FGFR3	Transcript	NM_000142.5
GC2_FGFR3	chr4:1 801 514_A/C	05/18	593	198	p.Gln198Ala	VUS	coding_sequence_variant	FGFR3	Transcript	NM_000142.5
GC2_FGFR3	chr4:1 801 535_AG/*	05/18	615	205	p.?	splice variant	coding_sequence_variant	FGFR3	Transcript	NM_000142.5
GC2_FGFR3	chr4:1 801 862_A/T	07/18	767	256	p.Gln256Leu	Not described	coding_sequence_variant	FGFR3	Transcript	NM_000142.5
GC2_FGFR3	chr4:1 805 564_G/A	13/18	1540	514	p.Ala514Thr	VUS	coding_sequence_variant	FGFR3	Transcript	NM_000142.5
GC2_FGFR3	chr4:1 805 565_C/CTGTCT	13/18	1541	514	p.Thr515Val fs*14	Not described	coding_sequence_variant	FGFR3	Transcript	NM_000142.5
GC2_FGFR3	chr4:1 805 760_C/T	14/18	1656	552	p.Tyr552Tyr	SNP (likely benin)	coding_sequence_variant	FGFR3	Transcript	NM_000142.5
GC2_FGFR3	chr4:1 805 929_G/A	14/18	1825	609	p.Ala609Thr	VUS	coding_sequence_variant	FGFR3	Transcript	NM_000142.5
GC2_FGFR3	chr4:1 806 060_A/G	15/18	1846	616	p.Arg616Gly	VUS	coding_sequence_variant	FGFR3	Transcript	NM_000142.5
GC2_FGFR3	chr4:1 806 085_T/A	15/18	1871	624	p.Leu624Gln	VUS	coding_sequence_variant	FGFR3	Transcript	NM_000142.5
GC2_FGFR3	chr4:1 806 154_A/C	15/18	1940	647	p.Tyr647Ser	VUS	coding_sequence_variant	FGFR3	Transcript	NM_000142.5
GC2_FGFR3	chr4:1 806 155_C/A	15/18	1941	647	p.647*	VUS	coding_sequence_variant	FGFR3	Transcript	NM_000142.5
GC2_FGFR3	chr4:1 806 157_A/C	15/18	1944	648	p.Tyr648Ser	Not described in cancer	coding_sequence_variant	FGFR3	Transcript	NM_000142.5
GC2_FGFR3	chr4:1 806 160_A/T	15/18	1946	649	p.Lys649Met	VUS	coding_sequence_variant	FGFR3	Transcript	NM_000142.5
GC2_FGFR3	chr4:1 806 165_ACGAC/A	15/18	1952-1956	651-652	p.Asn653Ala fs*5	Described once in skin carcinoma	coding_sequence_variant	FGFR3	Transcript	NM_000142.5
GC1_FGFR1	chr8:38 414 782_G/T (C>A)	14/18	1974	658	p.Thr658Thr	SNP VUS /Benin	coding_sequence_variant	FGFR1	Transcript	NM_023110.3
GC1_FGFR1	chr8:38 416 057_G/T (C>A)	13/18	1667	556	p.Pro556His	VUS	coding_sequence_variant	FGFR1	Transcript	NM_023110.3
GC1_FGFR1	chr8:38 418 232_C/T (G>A)	10/18	1426	476	p.Asp475Asn	VUS	coding_sequence_variant	FGFR1	Transcript	NM_023110.3
GC1_FGFR1	chr8:38 429 681_C/G (G>C)	03/18	358+1G>C	120	p.?	VUS	coding_sequence_variant	FGFR1	Transcript	NM_023110.3
GC1_FGFR2	chr10:12 150 0901_C/A	13/18	1486	496	p.Val496Phe	Vus	coding_sequence_variant	FGFR2	Transcript	NM_000141.5
GC1_FGFR3	chr4:1 801 623_C/T	06/18	619	207	p.Arg207Trp	VUS (described once in stomach tumor)	coding_sequence_variant	FGFR3	Transcript	NM_000142.5
GC1_FGFR3	chr4:1 805 771_A/G	14/18	1667	556	p.Glu556Gly	Not described	coding_sequence_variant	FGFR3	Transcript	NM_000142.5
GC1_FGFR3	chr4:1 806 164_GACGA/G	15/18	c.1951-1955 Del	651-652	p.Thr651_Thr652del fs*6AA	Not described	coding_sequence_variant	FGFR3	Transcript	NM_000142.5
GC1_FGFR3	chr4:1 806 167_G/A	15/18	1953	651	p.Thr651Thr	SNP VUS Benin	coding_sequence_variant	FGFR3	Transcript	NM_000142.5

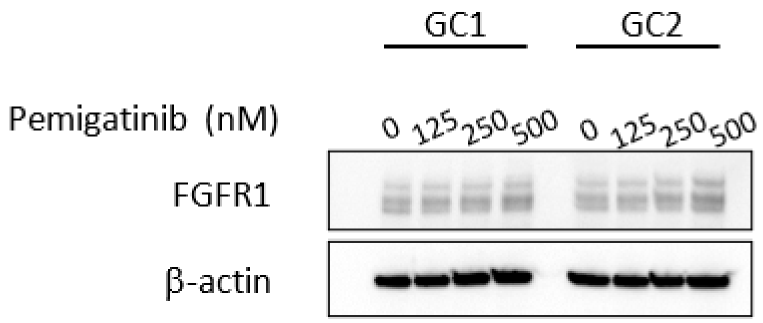

Figure S2. Western blot analysis of FGFR1 expression in differentiated glioblastoma stem cells. GC1 and GC2 were differentiated for two weeks before treatment with increasing concentrations of pemigatinib (125, 250, 500 nM) for 48 h. Untreated cells (0 nM) served as controls. β-actin was used as a loading control.

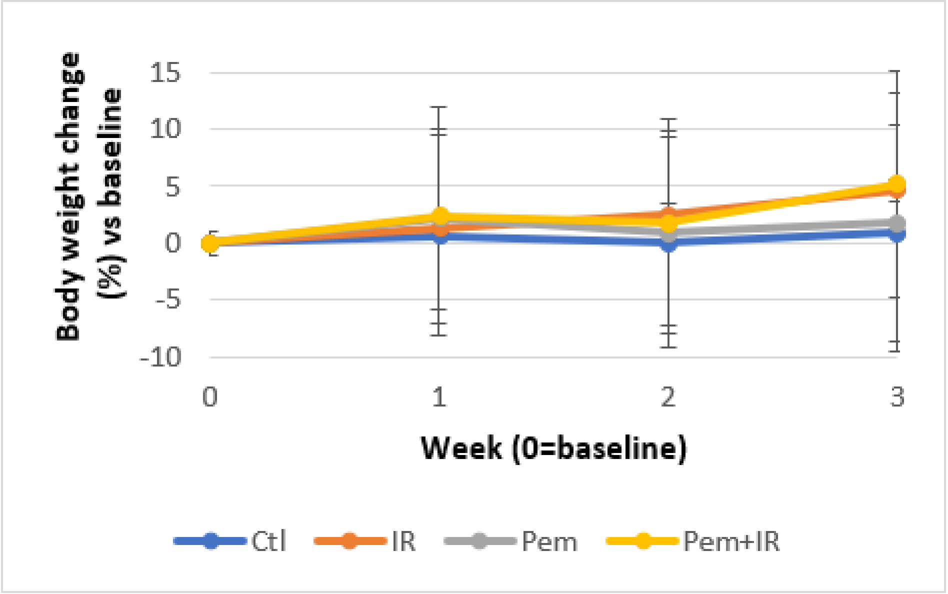

Figure S3. Body-weight trajectories (% change from baseline).
Mean ± SD of percent change in body weight from baseline (week 0) to week 3 for the CTL, IR, Pem, and Pem+IR cohorts. Error bars represent SD; at each time point, SDs were derived from the SDs of absolute body weights using error propagation.
